# Employees’ views on home-based, after-hours telephone triage by Dutch GP cooperatives

**DOI:** 10.1186/1865-1380-6-42

**Published:** 2013-11-04

**Authors:** Ramona Backhaus, Job van Exel, Antoinette de Bont

**Affiliations:** 1 Department of Health Services Research, Maastricht University, P.O. Box 616, 6200 MD, Maastricht, The Netherlands; 2 Institute of Health Policy and Management, Erasmus University Rotterdam, P.O. Box 1738, 3000 DR, Rotterdam, The Netherlands

**Keywords:** After-hours care, The Netherlands, General practitioners, Triage, Delegation, Professional, Remote consultation

## Abstract

**Background:**

Dutch out-of-hours (OOH) centers find it difficult to attract sufficient triage staff. They regard home-based triage as an option that might attract employees. Specially trained nurses are supposed to conduct triage by telephone from home for after-hours medical care. The central aim of this research is to investigate the views of employees of OOH centers in The Netherlands on home-based telephone triage in after-hours care.

**Methods:**

The study is a Q methodology study. Triage nurses, general practitioners (GPs) and managers of OOH centers ranked 36 opinion statements on home-based triage. We interviewed 10 participants to help develop and validate the statements for the Q sort, and 77 participants did the Q sort.

**Results:**

We identified four views on home-based telephone triage. Two generally favor home-based triage, one highlights some concerns and conditions, and one opposes it out of concern for quality. The four views perceive different sources of credibility for nurse triagists working from home.

**Conclusion:**

Home-based telephone triage is a controversial issue among triage nurses, GPs and managers of OOH centers. By identifying consensus and dissension among GPs, triagists, managers and regulators, this study generates four perspectives on home-based triage. In addition, it reveals the conditions considered important for home-based triage.

## Background

Since 2000, general practitioners (GPs) in The Netherlands have been delivering after-hours care in large-scale out-of-hours (OOH) services [1-3]. The GPCs were a first step to guaranteeing access to after-hours primary care [4]. In 2010, 128 GPCs organized urgent after-hours care for 16 million Dutch people [2]. The OOH centers reduced the number of evening and night shifts for GPs and also led to the introduction of a new professional: the triagist [5]. OOH triagists are specially trained nurses who determine the degree of urgency of a clinical problem presented by a patient on the telephone and decide whether the patient needs to see a GP [6]. OOH centers foresee a shortage of triagists [2]. To be more attractive employers, OOH centers are considering allowing triage nurses to work from home.

Prior research has shown that the quality of telephone triage depends upon the competencies of triage nurses, standardization of triage, and frequency of interaction between GPs and triage nurses. First, Derkx et al. [7] have defined the following three competencies for triage nurses: (1) medical knowledge, as one task of the triage nurse is to provide callers with advice about self-care and whether and when the caller should contact the OOH center again; (2) communication skills, as they have to ask questions and correctly interpret the information presented by the caller; (3) recording skills, as a triagist must be able to write a report that describes a telephone triage according to all medical and legal requirements.

Second, several authors [8-10] have discussed how standardization of procedures constrains the behavior of professionals and helps improve quality. For example, triagists must follow established steps to determine the medical urgency of a patient. Each step of the triage process is described and made visible. In addition, implicit knowledge of triage is made explicit and, consequently, controllable. Standardization could thus reduce the importance of mutual trust as the basis for cooperation between professionals [11].

Finally, Calnan and Rowe [11] showed that frequent interactions between GPs and other professionals contribute to quality, which can be the case for triage as well. Physical proximity and frequent communication between GPs and triagists help to overcome hierarchical differences that could prevent triage nurses from asking for feedback or sharing their knowledge with the GP.

To understand the conditions for home-based triage in more detail, we need to unpack the notion of trust. Trust is understood as a condition that triggers task delegation [11]. At this level of abstraction, trust can become an ill-defined residual. It needs to be specified. To specify trust, we introduce the notion of credibility, which is the quality of deserving to be trusted. According to Shapin [12], credibility is obtained differently between experts and the laity. In the expert context characterized by familiarity, professionals know each other very well and depend on each other’s work. In this context, not trust, but distrust and skepticism must be justified [12]. Consequently, professionals take each other’s claims at face value. In the lay context, professionals do not share the same level of expertise or values with lay people. Trust must then be justified by objective data. Quantification, such as performance data, may for instance help increase credibility between different expert groups, as it is a means to generate a shared discourse. Data then function as a response to criticism by others [13]. A relevant question is whether familiarity is the preferred basis for credibility. According to Shapin [12], we prefer to trust people we know. Quantification of trust is only an option when other methods do not work. All in all, Shapin [12] stresses that credibility is achieved in very diverse ways: it is about the relationship between the one who claims it and the one who is meant to believe.

The central aim of this research is to identify the different perspectives on home-based telephone triage among employees of Dutch OOH centers. The distinction Shapin [12] made between familiarity and laity will be central in our analysis. Home-based triage might affect the credibility of triagists as they no longer work at the same location as the GP. They become less familiar to the GP. As the nature and the frequency of interactions change between GPs and triagists, triagists might no longer be seen as part of the ‘clergy’ but to form the laity. As the triagists form the laity, they need new and more sources for credibility, such as procedures and performance data [12]. In addition, the attitude changes from trust to skepticism. The triagists will be asked whether they followed the procedures or met certain standards. Yet, Calnan and Rowe [11] showed that reliability and confidentiality as well as technical and communication skills are the most important determinants of trust between professionals, far more so than the outcome of performance measurements.

## Methods

We investigated the views of Dutch OOH center employees on home-based after-hours telephone triage using Q methodology, a method that combines aspects of qualitative and quantitative approaches for the systematic study of subjectivity [14,15]. Q methodology is still relatively novel to most health services researchers [16], but has gained popularity over the past 10 years, evidenced by rising numbers of publications on a variety of health and health care-related topics: for instance, attitudes and beliefs [17-19], treatment adherence [20,21], coping and adaptation [22-24], and professional views [25-27].

Q methodology rests on the assumption that a limited number of distinct subjective perspectives exist on any particular topic and that these can be measured and identified through systematic analysis. Participants are asked to rank and explain a number of items, usually statements of opinion, thereby revealing their subjective perspectives (view, preferences) on the topic. Next, assuming that similarity in ranking of items reflects similarity with perspective, the Q sorts are correlated and factor analyzed to identify a limited number of patterns in the rankings of items. These patterns are then interpreted and described as distinct perspectives. Q methodology can thus be used to identify and describe the perspectives that exist on a certain topic and for this purpose requires only a limited sample of purposively selected participants.

Conducting a Q study generally involves three consecutive steps: developing the statement set, identifying participants and conducting Q sort interviews, followed by analysis and interpretation of the Q sorts. These steps are described below for this study.

The statement set, usually called the Q sample, should represent the discourse around the topic of study, which can be obtained for instance through interviews or focus groups, or from academic or gray literature. In this study, databases from the Erasmus University Library, PubMed, Springerlink and Google Scholar were searched using combinations of the keywords ‘triage’ , ‘GP post’ , ‘GP cooperative’ , ‘home-based triage’ , ‘after-hours primary care’ , ‘urgent care’ , ‘Dutch primary care’ , ‘general practitioner’ , ‘restructure’ and ‘work’ (in English, German and Dutch). In addition, we studied related reports from the Association of Dutch OOH centers and the Dutch Ministry of Health, and recent newspaper articles. Using these sources, we developed a topic list for semi-structured interviews with stakeholders. In March 2012, we tape-recorded and subsequently transcribed ten interviews with trainers of triagists (2), managers of OOH centers (2), triagists (2), a GP, a quality control worker, an inspector from the Dutch Health Care Inspectorate and a policy maker from the Dutch Ministry of Health. Analysis revealed eight recurring themes in perspectives on home-based telephone triage: quality, tasks, autonomy, trust, communication, collegiality, privacy and standardization. Statements collected from the literature and the interviews were categorized according to these themes. In an iterative process involving all three authors, we deleted duplicate statements, merged similar statements and selected a draft set of 36 statements. We held pilot interviews with two triagists to check for completeness and intelligibility of the statement set and other interview materials.

**Figure 1:**
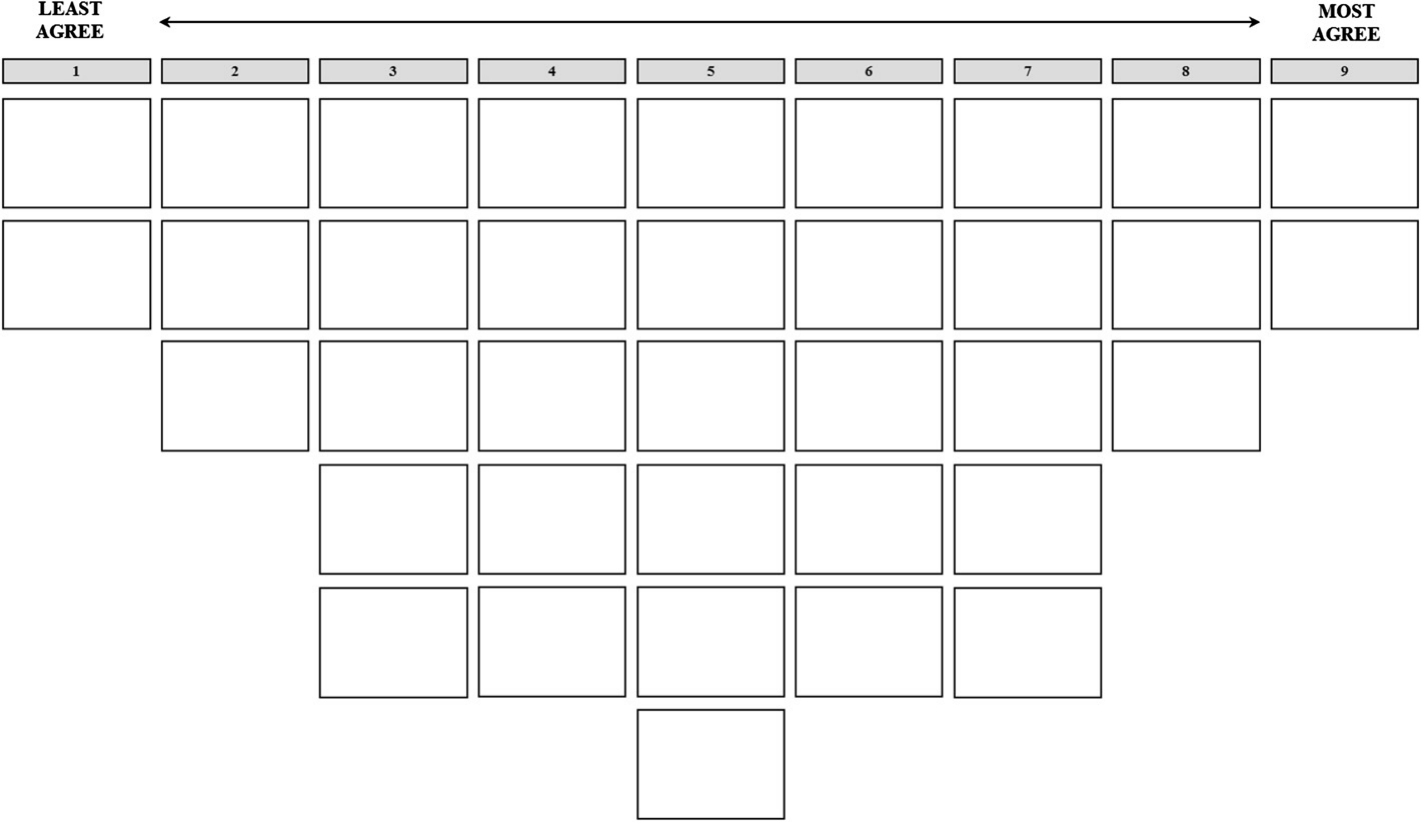
Score sheet for Q sort.

Between March and June 2012, we conducted 77 Q sort interviews with employees of OOH centers who were potentially concerned with home-based telephone triage: 33 triagists and GP assistants, 8 GPs, 20 managers of OOH centers and 16 others, including triagist trainers, quality control staff and human resources staff. We recruited the participants through selected OOH centers and the Dutch Association of OOH centers. We asked participants to read the 36 statements and sort them into three categories: agree, disagree and neutral. Next, we asked them to reread the statements they agreed with and place them on the score sheet according to extent of agreement (Figure 1). They then repeated the procedure for the statements they disagreed with and those left in the neutral pile. After the participants had finished the Q sort, they were asked to check it and make changes if needed. Finally, they were asked to explain their Q sort, with emphasis on the statements placed at the extremes of the score sheet.

We used the dedicated software package PQMethod 2.11 to conduct the analysis [28]. Data were analyzed with by-person factor analysis, using conventional factor extraction and rotation techniques for Q analysis. The data supported a maximum of five factors. We inspected the three-, four- and five-factor solutions. For each factor we computed an idealized Q sort, representing how a person loading 100% on that factor (i.e., holding that specific view) would have ranked the 36 opinion statements. A statement is characterizing for a factor when its score on the factor is +4, +3, -3 or −4 in the idealized Q sort. However, it is possible that a statement may stand for more than one opinion, as people with different viewpoints may agree on certain aspects. A statement differentiates for a factor when its score on the factor is statistically significantly different from its score on the other factors. In other words, a statement differentiates when it is positioned clearly differently in the idealized Q sort. In the final step of the analysis the factor interpretation was checked with the explanations given by participants during the Q sort interview.

The four-factor solution was selected as the preferable solution for the data because it had a clearly interpretable account for each factor, which was consistent with the written and verbal comments the participants provided while defining the factors to complete the Q sort. Together the four factors explained 45% of the total variance in the Q sorts, which is at the low end of a fairly typical range (between 45 and 50%) observed in Q studies. The correlation between factors ranged between 0.23 and 0.59. Table 1 presents the idealized Q sorts for each of the four factors, highlighting the distinguishing statements. Each factor represents a distinct perspective on home-based triage by employees of OOH centers, described in detail in the Results section.

**Table 1:** Factor array

**Statement**	**Factor**			
	**1**	**2**	**3**	**4**
1. Home-based triage is desired as backup during unexpected busy hours at an OOH center	+4**	−2	−2	+3**
2. If triagists want to work from home, it should be possible	0**	−4**	−2	−2
3. It is important that a GP and a triagist work in the same location	−2**	+3**	0	−1
4. The possibility for triagists to work from home is not an option for dealing with staff shortages	−1**	0	0	+1
5. If triagists work from home, it will not be possible to give adequate feedback on their functioning	−1	+4**	−1	+2**
6. If triagists work from home, the quality of triage will decrease	−2	+1**	−2	−2
7. Due to the distractions of a busy household, triagists cannot work well at home	+2*	0	+1	−1**
8. If triage is home-based, then mutual trust between triagists will decrease	0	−1	−3**	+1**
9. Triagists can execute telephone triage better than GPs	+3	+2	+2	+1
10. Work experience at an OOH center is important for executing home-based triage	+3	+2**	+3	+4**
11. Home-based triage can only occur if there is mutual trust between the triagist and the attendant GP	+2*	−2**	+1*	+3**
12. Home-based triage is possible for all the tasks telephone triagists have	+1**	−3*	−3*	−2**
13. A prerequisite for home-based triage is that triagists also take on shifts at the OOH center	0	0	+2**	+4**
14. GPs’ trust in triage decreases when they do not know the triagist	+2	+1	+2	+2
15. Home-based triage can only take place during the day	−4	−2**	−4	−4
16. The quality of triage is harder to measure when triagists work from home	−2**	+2**	−1	−1
17. Home-based triage extends the length of the triage process	−2	−1	−1	−3
18. Home-based triage leads to increased differences in how triagists do their work	0	0	0	0
19. Triagists who work from home will ask the GP for advice more often	−2	+1*	−1*	−2
20. Patients’ trust in OOH services will decrease if triage is home-based	0	−1	0	−3**
21. Mutual communication decreases when triagists are home-based	+2	+2	+1	+2
22. Poorly functioning home-based triagists will not be detected (at all or later) than those working at an OOH center	−1**	+1	+1	+2*
23. Employment of a telephone physician is a prerequisite to offering home-based triage	+1**	−2**	+4**	0**
24. Home-based triage leads to a decrease in collegiality	0	+2	+3*	+2*
25. More standardization is necessary if triagists are to work from home	−1	−1	0	0
26. In a home-based situation, the privacy of patients’ medical data can be protected only inadequately	+1	+4**	+2	−2**
27. If triagists work from home the task of the GP will change	−3	0	−3	−1
28. Shift changes between GPS will be complicated if triage is home-based	−1	+1**	−2**	0
29. If triage is home-based the work pressure on GPs increases	−3*	−1*	−2*	−1*
30. A separate workplace at home is a requirement for home-based telephone triage	+4	0**	+4	+1**
31. Primarily for OOH centers with large catchment areas, triagists should be able to work from home	+1**	−3	−1**	−3
32. The triagist should be able to see all [digital] patient dossiers at home	−3	−3	+3**	0**
33. Triagists will miss out on learning from each other if their work is home-based	+3	+3	+1**	+3
34. If triage is home-based, the number of doctors on duty should be reduced	−4	−4	−4	−4
35. A quiet workplace at home is more suitable for offering triage than the busy environment at a GPC	+1**	−2	0	0
36. Cooperation between triagists and GPs will decrease if triage is home-based	+2	+3	+2	+1*

Note: Higher scores indicate stronger agreement with the statement. Statement distinguishing for factor: *p < 0.05; **p < 0.01.

## Results

We distinguished four views on home-based telephone triage in after-hours care among employees of Dutch OOH centers.

### Home-based triage is an option for unexpected busy hours

This first view does not favor home-based telephone triage, but accepts it as potential backup for unexpected busy hours (1: +4). The employees defining this view agree that home-based triage can only take place when there is mutual trust between the triagists and the GP on duty and they know each other well (1: +2). A human resources employee explained: “Home-based triage could be a solution to decrease work pressure in busy hours. Our telephone system offers this possibility but we’ve never tried it out. Working from home, triagists who could help out would be available more quickly, as they do not have to travel to the OOH center first.” Thus, availability of service in after-hours care is put at the forefront. The possibility to work from home is seen as an alternative for triage at the OOH center in order to deal with employee shortages (4: -1).

More than others, the employees of OOH centers defining this view agree that triagists conduct telephone triage better than the GPs do (9: +3). They disagree that monitoring the quality of triage becomes harder if triagists work at home (16: -2) and share the viewpoint that the work pressure of GPs would not increase as a result (29: -3).

In sum, they do not see the necessity of triagists and GPs working at the same location (3: -2). In contrast, the proponents of this view do not regard the availability of full patient data in the home setting as a requirement (32: -3). One location manager argued that “offering patient data in the home setting would lead to violations of privacy” and therefore “only the information that is necessary for the triage process should be available.” In addition, participants sharing this view were the only ones who think that home-based triage is possible for all tasks a triagist has (12: +1). Holders of this view were the only ones neutral about making home-based triage possible if triagists want to work from home (2: 0).

### Home-based triage is not an option

Strong opposition against home-based telephone triage characterizes the second perspective. One manager expressed his view by saying, “Total rubbish! Triage will never take place in a home setting.” The employees who hold this perspective criticize home-based triage as it provides very few opportunities for feedback (5: +4). A triagist explains: “When we work at the same location, we learn from each other’s telephone calls.” They are the only ones who stress the importance of a shared location (3: +3). More than others, they expect to see less teamwork between triagists and GPs if triagists worked from home (36: +3). At the same time, they expect home-based triagists will ask the GP for advice more often (19: +1), something they regard as a negative.

Opposition to home-based triage is associated with a number of fears not shared by the other perspectives on home-based triage. It is the fear that privacy is at stake in the home setting (26: +4), that it will be hard to monitor the quality of triage in the home setting (16: +2) and that it complicates the rotation of shifts at the OOH centers (28: -1). In general, they fear that home-based triage will lead to a lower triage quality (6: +1).

This is the only view to disagree with the statement that home-based triage can only take place when there is mutual trust between the triagists and the GP on duty (11: -2).

### Controllability is an option for home-based triage

In this third view, home-base triage is believed to be feasible if certain conditions are set and met. “Home-based triage could work as long as the computer system is secure so that privacy is not at stake, if it offers good quality and if it enables good communication and coordination between triagists and (telephone) GPs.” The first condition employees defining this view put forward is the employment of a telephone GP (23: +4). One triagist described the telephone GP as responsible for “control and overview.” Furthermore, they considered a separate workplace at home (30: +4) and the availability of full patient information (32: +3) necessary. A triagist explained, “Triagists should have access to patient records, especially in case a patient has called before or because of a complication.” Nevertheless, the proponents of this view fear that privacy could be at stake in the home setting (26: +2).

### Familiarity is a prerequisite for home-based triage

In this fourth view, familiarity with both the organization and the team is considered central to good triage. More than in the other views, work experience at an OOH center is seen as a requirement for triagists to work from home (10: +4). One triagist stressed that “having worked at an OOH center is necessary to understand the system, get to know the procedures and your colleagues and show that you can take on the responsibility of working from home.” A GP added that work experience at an OOH center is necessary to “know what to do with the information obtained through the triage process.” Employees defining this view think that home-based triagists should also work at the OOH center (13: +4). One triagist argued that “good quality home-based triage can only be offered when the triagist works also regularly at the OOH center. Then the triagist knows the local arrangements and knows her colleagues.” A GP stressed that working regularly at the OOH center would increase collegiality between employees. “Collegiality decreases as you would not see each other often, and that makes it far more difficult to give feedback. I can imagine that not seeing each other regularly would make it even harder to discuss a feeling of unease with each other.” Accordingly, this view is the only one expressing fear that mutual trust between the triagist and the GP would decrease (8: +1). More than in other views, mutual trust between the triagist and the GP is regarded as a requirement for home-based telephone triage (11: +3).

## Discussion

The study distinguished four views on home-based triage. Three (1, 3 and 4) generally favor home-based triage, two (1 and 3) highlight some concerns and conditions, and one (2) opposes home-based triage out of a concern for quality. This study also revealed difference in the sources that support the credibility of triage nurses. Perspectives 2 and 4 both stress familiarity as the importance of being well known to the triagist or the GP with whom they work. However, while perspective 2 stresses the importance sharing the same location to give informal feedback, perspective 4 stresses the importance of tacit knowledge as local routines. Only when triage nurses work in the after-hours service will they learn the local routines. In contrast, the two perspectives that advocate home-based triage are significantly different in what they perceive as the most important sources of credibility. Views 1 and 3 stress the importance of monitoring as the most important source for credibility. Trust develops as triagists show they have followed the procedures and met the standards. Thus, rather than standpoints for or against making home-based triage possible, this study revealed diverse viewpoints related to various opportunities or concerns, and different sources of trust. With the use of Q methodology, we generated a more detailed understanding of the discussion around home-based triage in Dutch OOH centers, including the conditions considered important, and the role of trust.

A weakness of this study was participant selection. Beforehand we were unable to identify clear recruitment criteria, and therefore we ended up conducting an above average number of interviews for a Q methodology study before we felt we had reached saturation point. As home-based triage is still rare and this study was conducted to support the development of a pilot project for home-based triage, participants lacked experience in the subject. Viewpoints might thus change in time, when employees of OOH centers have gained experience. Replication of this study would then be interesting.

As the introduction pointed out, the evidence is inconclusive as to whether mutual trust – based upon familiarity – becomes less important if the work of triage nurses can be standardized [7-10]. In other words, it is questionable whether frequent interactions and physical or social proximity become less important for collaborative work that can (partly) be done from home. The literature about triage lists different sources of credibility: frequent interactions, skills, reliability, confidentiality, standardization and outcomes of performance measurements. This study differentiates two sources of credibility – frequent interactions and skills –from the other sources. The first two sources presume familiarity as the basis of trust; the other sources presume laity as the basis on which trust is built. This difference between familiarity and laity as sources of credibility [12] raises the question whether the different sources for credibility that are listed in the literature [7-10] can indeed be mixed.

The results of this study have implications for both clinicians and policy makers. As Shapin [12] pointed out, we prefer to collaborate with people we know and, because we know them, we can avoid asking questions about the quality of each other’s work. At the same time, we – both policy makers and clinicians – develop tools to ask questions to control the quality of work. That happened in this study about triage, but we see a similar dilemma in, for example, initiatives to enhance patient safety. This study shows that procedures for quality management interact with the way we prefer to trust each other [12]. The study clearly differentiates the two manners of credibility. It raises the question whether we can work with the people we know without asking questions and at the same time allow questions to be asked to check whether the procedures were followed.

## Conclusion

In conclusion, home-based telephone triage is a controversial issue. This study revealed that there is strict opposition to it, out of concern for the quality of triage, and that advocacy comes with different concerns, conditions and sources for building and maintaining trust between professionals. Although the study was conducted to support the development of a pilot project for home-based triage, the strict opposition to it hampers the implementation of a pilot project.

## Competing interests

The authors declare that they have no competing interests.

## Authors’ contributions

RB designed the study, collected the data, analyzed the data and drafted the manuscript. AB revised the study design, interpreted the results of the data analysis and revised the manuscript. JE revised the data analyzed and revised the manuscript. All authors read and approved the final manuscript.
